# Lupus Cured by Arsenic Internally

**Published:** 1885-07

**Authors:** 


### LUPUS CURED BY ARSENIC INTERNALLY.

*New Orleans Medical and Surgical Journal:*

"Mr. Jonathan Hutchinson published in the *British Medical Journal* an account of a case of lupus erythematosus cured by the internal administration of arsenic. The remedy was pushed until physiological effects, red eyes and an attack of "shingles" appeared. Mr. Hutchinson is not very sanguine, however, and seems to regard the case as exceptional."

